# Soluble Urokinase Plasminogen Activator Receptor (suPAR) as a Biomarker of Erectile Dysfunction in Diabetic Patients

**DOI:** 10.3390/jcm14124029

**Published:** 2025-06-06

**Authors:** Osman Erinc, Ozgur Yilmaz, Tacettin Yekta Kaya, Murvet Algemi, Murat Akarsu

**Affiliations:** 1 Department of Internal Medicine, Kanuni Sultan Suleyman Training and Research Hospital, Istanbul 34303, Turkey; 2 Department of Urology, Kanuni Sultan Suleyman Training and Research Hospital, Istanbul 34303, Turkey; 3 Department of Clinical Biochemistry, Kanuni Sultan Suleyman Training and Research Hospital, Istanbul 34303, Turkey

**Keywords:** soluble urokinase plasminogen activator receptor (suPAR), erectile dysfunction, diabetes mellitus

## Abstract

**Background/Objectives:** Erectile dysfunction (ED) is a common complication of diabetes mellitus (DM), largely attributable to vascular, neurological and metabolic dysfunctions. Soluble urokinase plasminogen activator receptor (suPAR) is a biomarker of systemic inflammation and endothelial dysfunction, both of which play key roles in ED pathophysiology. This study aimed to evaluate the relationship between serum suPAR levels and ED in patients with type 2 DM, assessing its potential as a biomarker for early detection. **Methods:** This prospective, cross-sectional study included 127 male patients with type 2 DM and 46 healthy controls. Erectile function was assessed using the International Index of Erectile Function-5. Patients were divided into three groups: controls, diabetic patients without ED (DM-NoED) and diabetic patients with ED (DMED). Serum suPAR levels were measured via ELISA. Statistical analyses included Kruskal–Wallis tests, Dunn’s post hoc comparisons and ROC curve analysis to evaluate diagnostic performance. **Results:** Serum suPAR levels were significantly elevated in the DMED group compared to both the DM-NoED and control groups (*p* < 0.001). The median suPAR levels were 107.9 pg/mL (controls), 130.3 pg/mL (DM-NoED) and 218.7 pg/mL (DMED). ROC analysis revealed an AUC of 0.836 in distinguishing DMED from DM-NoED with 87.5% sensitivity and 79.2% specificity. **Conclusions:** Elevated serum suPAR levels are significantly associated with ED in men with type 2 DM, independent of glycemic control and conventional cardiovascular risk factors. These findings suggest that suPAR may be a promising biomarker for the early detection and risk assessment of ED in diabetic patients. Future prospective studies are needed to confirm its clinical utility.

## 1. Introduction

Erectile dysfunction (ED) is a common condition in diabetes mellitus (DM), defined as a man’s inability to attain or sustain a sufficient erection during sexual intercourse [1,2]. ED is a multifaceted disorder arising from a complex interaction of vascular, neurological, hormonal and psychological elements.

During sexual arousal, activation of the parasympathetic nervous system stimulates the release of nitric oxide (NO) from both endothelial cells and non-adrenergic, non-cholinergic neurons. NO activates guanylate cyclase, increasing intracellular cyclic guanosine monophosphate (cGMP) levels. Elevated cGMP reduces intracellular calcium concentrations, leading to relaxation of the cavernosal smooth muscles. This relaxation compresses the subtunical venules, thereby occluding venous return and resulting in an erection. The phosphodiesterase 5 (PDE5) enzyme regulates cGMP-dependent penile erection by stimulating the hydrolysis of cGMP [3]. Disruptions at any point—whether resulting from vascular, neurologic and hormonal imbalances or psychological stress—may cause ED [4,5,6,7].

The onset of ED in diabetic patients occurs earlier and more severely, attributable to a combination of vascular, neurological and metabolic dysfunctions [8]. Persistent hyperglycemia plays a pivotal role that leads to endothelial dysfunction, oxidative damage, autonomous neuropathy and smooth muscle abnormalities [9]. Reduced NO bioavailability due to endothelial dysfunction, a hallmark of diabetes, hinders the relaxation of cavernous smooth muscle and vasodilation [10,11,12]. Furthermore, hyperglycemia encourages the formation of advanced glycation end-products, which worsens penile artery atherosclerosis, inflammation and vascular stiffness [13,14]. Diabetic neuropathy exacerbates ED by impairing autonomic and somatic neurons associated with the erectile response [15,16]. Also, diabetes is related to hypogonadism, which causes problems with libido and erectile function [17]. In diabetics, multifactorial aspects aggravate ED, rendering it more severe and less susceptible to standard therapies such as PDE5 inhibitors [18].

Soluble urokinase plasminogen activator receptor (suPAR) represents the soluble variant of the membrane-bound urokinase plasminogen activator receptor (uPAR), which is a glycosylphosphatidylinositol-anchored protein found on immune, endothelial and smooth muscle cells. uPAR regulates plasminogen activation and extracellular matrix degradation, which are vital for tissue remodeling, immune response and cell migration. SuPAR is released into the circulation upon cell activation and acts as a systemic biomarker for immune system activation, prolonged inflammation and endothelial dysfunction. In addition to its involvement in fibrinolysis and angiogenesis, suPAR impacts integrin signaling and plays a role in immune cell trafficking, particularly upon infection or chronic inflammation [19]. SuPAR has emerged as a promising biomarker reflecting systemic inflammation, immune activation and vascular pathology across a broad spectrum of clinical conditions. Raggam et al. conducted a prospective study involving 902 patients with systemic inflammatory response syndrome SIRS and demonstrated that early measurement of serum suPAR levels is predictive of mortality. Moreover, suPAR outperformed traditional biomarkers such as C-reactive protein (CRP), interleukin-6 and procalcitonin in reflecting systemic inflammation [20]. SuPAR has also gained attention in infectious diseases, emphasizing its utility in acute inflammatory syndromes [21]. In pediatric endocrinology, Sherif et al. demonstrated elevated suPAR levels in children with type 1 diabetes, correlating with the presence of microvascular complications, thereby highlighting its relevance in diabetic vasculopathy [22]. Similarly, Wu et al. showed that suPAR levels were significantly associated with the severity of diabetic nephropathy and other biopsy-proven kidney diseases, reinforcing its role as a marker of renal involvement and disease progression [23]. Collectively, these findings underscore suPAR’s clinical potential as a dynamic indicator of systemic inflammatory burden and organ damage.

SuPAR has been associated with endothelial dysfunction, increased vascular permeability and oxidative stress in cardiovascular and metabolic disorders, which are all key factors in the development of diabetic vascular complications, such as nephropathy, retinopathy and ED. SuPAR levels correlate with the severity of coronary artery disease and cardiovascular mortality, highlighting its significance in vascular health [24,25,26,27,28]. In a study conducted by Corban MT. et al., it was shown that patients with early coronary atherosclerosis exhibited elevated local coronary production of suPAR, which could be associated with both epicardial and microvascular endothelial dysfunction [29]. Furthermore, suPAR has been linked to glomerular and podocyte damage, leading to impaired renal function, and is associated with diabetes duration and complications, independent of other risk factors [30].

Given the close interplay between inflammation, endothelial dysfunction and erectile physiology, investigating suPAR’s utility as a biomarker in diabetic patients with ED could provide novel insights. To the best of our knowledge, no studies have directly examined the association between suPAR and ED. This article aims to evaluate the relationship between suPAR levels and erectile dysfunction in patients with diabetes mellitus, examining whether suPAR could be a potential biomarker for ED.

## 2. Materials and Methods

### 2.1. Study Design and Setting

This prospective, cross-sectional study included 127 male patients with type 2 DM and 46 male participants without any known disease or complaint, aged between 18 and 80 years, who were referred to internal medicine outpatient clinics of our hospital. A detailed medical history was obtained and a physical examination (weight, height, blood pressure), including an endocrinological evaluation, was performed. After explaining the purpose and details of the study and taking written consent, they were referred to a urologist with over 10 years of experience. All participants underwent urologic examination, and their sexual health was assessed via the clinician-administered International Index of Erectile Function-5 (IIEF-5) questionnaire. Patients with IIEF-5 scores <22 were included in the erectile dysfunction group, while those with scores ≥22 were classified as having no ED.

Participants were split into three groups: Group 1 (*n* = 46) was a control group that consisted of healthy individuals, Group 2 (*n* = 54) consisted of diabetic patients without ED and Group 3 (*n* = 73) consisted of diabetic patients with ED. Patients with type 1 DM, hyperprolactinemia, hypogonadism or thyroid disorders, or who had undergone major pelvic surgery; patients with a past history of total or transurethral prostatectomy, prostate malignancy or benign prostate hyperplasia; patients with severe renal, hepatic, cardiovascular or neurologic disease; patients with acute or chronic infection; patients who were using medications that may cause erectile dysfunction—such as certain antihypertensive agents (e.g., beta-blockers, thiazide diuretics), antidepressants (e.g., selective serotonin reuptake inhibitors), antipsychotics or hormonal therapies (e.g., antiandrogens); and patients with a history of psychiatric disorders were excluded from the study. These strict exclusion criteria aimed to obtain a homogeneous study population.

### 2.2. Erectile Function Assessment

The IIEF-5 questionnaire includes five questions, each with a maximum score of five, yielding a total maximum score of 25, which evaluates an individual’s ability to attain and sustain an erection. An IIEF-5 score <22 indicates ED, while an IIEF-5 score ≥22 indicates normal erectile function. ED severity was categorized as severe (5–7), moderate (8–11), mild to moderate (12–16) or mild (17–21) [31].

### 2.3. Blood Sampling and Biochemical Analysis

Blood was taken from the subjects’ cubital vein and collected in serum and plasma containers. The staff of the laboratory were functionally blinded to the aims of the study. The samples were centrifuged at 2000 rpm for 20 min at 4 °C. Subsequently, the supernatant was collected and kept at −80 °C until analyses were conducted. Fasting plasma glucose, creatinine, alanine aminotransferase, albumin, lipid profile (enzymatic colorimetric method) and CRP (immunoturbidimetric method) were measured using a Roche Cobas 8000 c 702 analyzer (Roche Diagnostics, Mannheim, Germany). Glycated hemoglobin (HbA1c) was assessed via high-performance liquid chromatography using an ARKRAY/ADAMS HA-8180V (ARKRAY Inc., Kyoto, Japan). Total testosterone, total prostate-specific antigen (PSA), luteinizing hormone (LH), follicle-stimulating hormone (FSH), prolactin, thyroid-stimulating hormone (TSH) and insulin were analyzed by electrochemiluminescence on a Roche Cobas E801 analyzer (Roche Diagnostics, Mannheim, Germany).

### 2.4. Serum suPAR Measurement

Venous blood samples were collected for suPAR and were centrifuged, and the sera were separated into Eppendorf tubes and stored at −80 °C until analyses. A commercially available double-antibody sandwich technique enzyme-linked immunosorbent assay kit (Human suPAR, Sunred Biological Technology Co., Baoshan, Shanghai, China), validated for 5 pg/mL and 1000 pg/mL, was used according to the manufacturer’s protocol. Measurements were performed using an ELX800DA device (Diagnostic Automation Inc., Woodland Hills, CA, USA) and analyzed via KC Junior software version 1.41 (Agilent Technologies, Santa Clara, CA, USA). A single-blinded technician conducted all of the measurements.

### 2.5. Outcomes

The primary outcome of this study was to evaluate the association between serum suPAR levels and the presence of ED in diabetic patients. The secondary outcome included evaluating the diagnostic accuracy of suPAR for ED through receiver operating characteristic (ROC) analysis.

### 2.6. Statistical Analyses

Statistical analyses were performed using SPSS version 27.0 (IBM Corp., Armonk, NY, USA). The data distribution was checked using the Kolmogorov–Smirnov test, and the results indicated that they did not have a normal distribution. Due to this distribution, the values are expressed as the median and interquartile range (IQR). Categorical parameters are indicated as percentages. Therefore, the Kruskal–Wallis test was used for group comparisons with a Dunn’s post hoc test. The Chi-square test was used in the analysis of qualitative independent data. ROC analysis was performed, and the optimal cut-off value was calculated using the Youden index. A *p*-value below 0.05 was deemed statistically significant.

## 3. Results

A total of 173 male participants were analyzed, including 46 healthy controls, 54 diabetic patients without ED (DM-NoED) and 73 diabetic patients with ED (DMED). While age showed an increasing trend across groups, this did not reach statistical significance (*p* = 0.055). Body mass index was significantly elevated in both diabetic groups compared to the controls (*p* < 0.001). Diabetes duration was significantly longer in the DMED group (14 (6–19.5) years) than in the DM-NoED group (7 (3–14) years, *p* < 0.001). Systolic and diastolic blood pressure did not differ significantly across the groups. Table 1 summarizes the demographic, anthropometric and clinical data across the study groups.

As shown in Table 2, serum suPAR levels differed significantly among all groups (*p* < 0.001). The median levels were 107.9 pg/mL in the controls, 130.3 pg/mL in DM-NoED and 218.7 pg/mL in DMED. Post hoc analyses confirmed that suPAR levels in the DMED group were significantly higher than both those in DM-NoED and the controls (*p* < 0.001).

The IIEF-5 scores were significantly lower in the DMED group (11 (6–15)) than in the DM-NoED (24 (23–25)) and control groups (23 (22–24)) (*p* < 0.001). ED severity in the DMED group was distributed as follows: 20.5% mild, 26.0% mild to moderate, 22.0% moderate and 31.5% severe. Despite these findings, comorbidities including ischemic heart disease, hypertension and hyperlipidemia did not differ significantly between the DM-NoED and DMED groups.

Fasting blood glucose, HbA1c and HOMA-IR values were significantly elevated in both diabetic groups compared to the controls (all *p* < 0.001); however, there were no statistically significant differences between the DM-NoED and DMED groups for these parameters.

Lipid profiles, testosterone, PSA, prolactin, FSH, LH, TSH, renal and hepatic function parameters and inflammatory markers, including CRP and erythrocyte sedimentation rate (ESR), did not differ significantly among the three groups (all *p* > 0.05).

**Figure 1:**
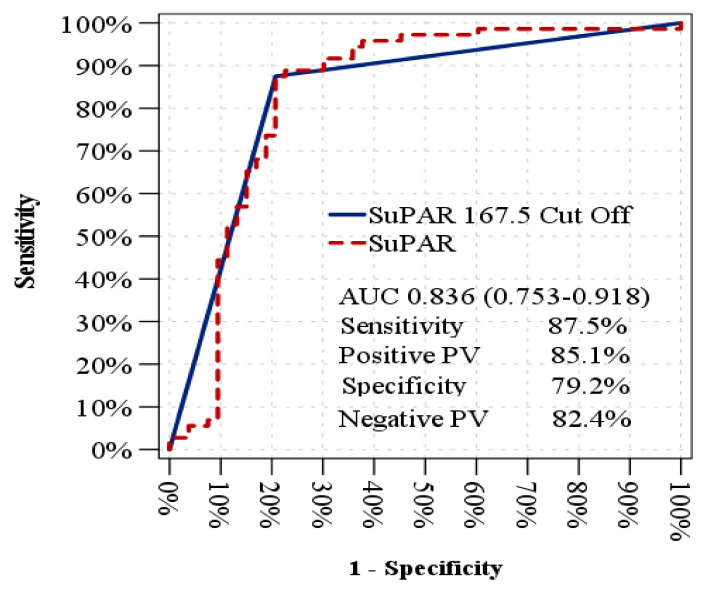
An analysis using receiver operating characteristic (ROC) curves to predict erectile dysfunction in diabetic patients.

ROC curve analysis revealed that the distinction between diabetic patients with and without erectile dysfunction (DMED vs. DM-NoED) based on serum suPAR levels was statistically significant, with a cut-off value of 167.5 pg/mL, yielding an area under the curve (AUC) of 0.836 (95% CI: 0.753–0.911), sensitivity of 87.5%, specificity of 79.2%, a positive predictive value of 85.1% and a negative predictive value of 82.4% (Figure 1).

**Figure 2:**
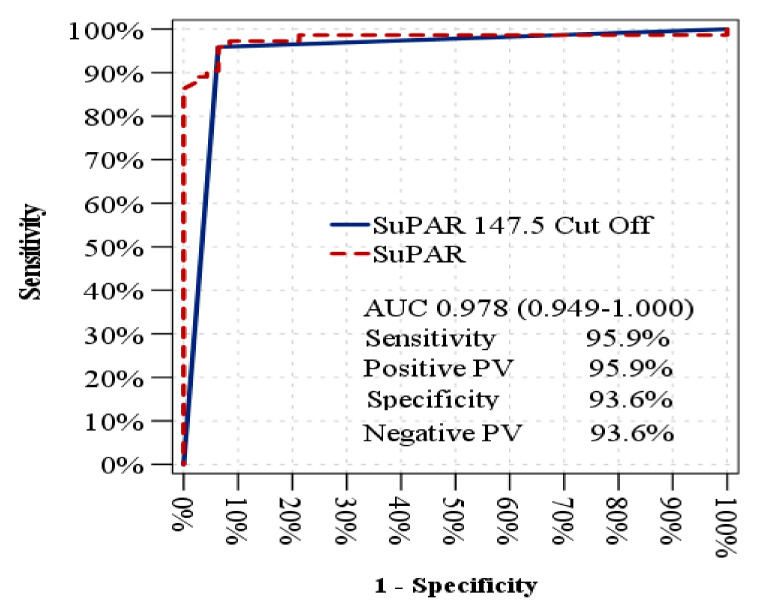
ROC analysis to distinguish DMED patients from healthy controls.

Furthermore, suPAR also successfully distinguished DMED patients from healthy controls with an AUC of 0.948 (95% CI: 0.899–0.996) at a cut-off of 147.5 pg/mL (sensitivity: 95.9%; specificity: 93.6%) (Figure 2).

## 4. Discussion

This study demonstrates a significant association between elevated serum suPAR levels and the presence of ED in male patients with type 2 diabetes mellitus. The findings show that suPAR concentrations were markedly higher in the DMED group than in both the DM-NoED group and healthy controls. Importantly, this difference was observed despite no significant intergroup variation in glycemic indices (HbA1c, fasting glucose, HOMA-IR), hormonal parameters (testosterone, LH, FSH), lipid profiles or conventional inflammatory markers such as CRP and ESR.

In this study, no significant differences were detected between the DMED and DM-NoED groups regarding glycemic control parameters such as HbA1c, fasting plasma glucose and HOMA-IR. However, the fact that serum suPAR levels were found to be markedly higher in the DMED group despite this is noteworthy. This finding may reveal the potential of suPAR to more sensitively reflect the underlying pathophysiological processes—particularly chronic inflammation and subclinical endothelial dysfunction—behind erectile dysfunction in diabetic men, independent of glycemic control status. In this context, suPAR may be considered in clinical practice as a complementary biomarker to glycemic parameters for the early diagnosis of ED and vascular risk assessment in diabetic individuals.

A link between suPAR and ED in diabetes may be biologically plausible, given suPAR’s known roles that could potentially support this association. suPAR is shed into the circulation upon immune cell activation and may reflect immune system engagement and low-grade inflammation [32,33]. In a recent review, Rasmussen et al. emphasized that suPAR may represent a potential gold-standard biomarker of systemic chronic inflammation due to its stable association with immune activation and inflammatory burden [19]. Moreover, Hindy et al. reported that elevated suPAR levels modulate monocyte activation and trafficking, thereby promoting vascular inflammation and atherosclerosis [34]. Consistent with this perspective, our study found significantly higher suPAR concentrations in the DMED group compared to the DM-NoED group, despite the absence of significant differences in lipid, hormonal and conventional inflammatory markers. This suggests that suPAR may reflect chronic inflammation and subclinical endothelial dysfunction not captured by routine laboratory indices.

The strong performance of suPAR in ROC analysis underscores its potential as a predictive biomarker. An AUC of 0.873 demonstrates discriminatory power, aligning with previous literature linking suPAR to cardiovascular and renal complications in diabetes [35]. According to Velissaris et al.’s review, elevated suPAR levels are independently associated with adverse outcomes and mortality in clinical scenarios such as acute coronary syndromes and congestive heart failure. Among various biomarkers used for clinical entities with underlying inflammatory pathophysiology, including cardiac diseases, suPAR stands out as a novel and promising indicator for prognostic risk stratification in cardiac patients [36]. A study conducted by Curovic et al. linking suPAR to diabetic complications revealed that elevated suPAR levels were significantly correlated with cardiovascular events and a decline in the estimated glomerular filtration rate [37]. Similarly, Mortberg et al. showed that suPAR was independently associated with the composite outcome of myocardial infarction, ischemic stroke, heart failure or death [38].

This study has some limitations. First, its cross-sectional design prevents assessment of temporal or causal relationships. Longitudinal studies are required to determine whether elevated suPAR levels precede ED onset or track with disease progression. Second, although rigorous exclusion criteria were applied, residual confounding unmeasured variables (e.g., psychosocial stress, physical activity) cannot be excluded. Third, the relatively small sample size and the single-center nature of the study may limit the generalizability of our findings and necessitate confirmation in larger, multicenter cohorts.

In conclusion, serum suPAR levels are significantly elevated in diabetic patients with ED. suPAR showed significant diagnostic performance in identifying diabetic men with ED, independent of glycemic control or classical cardiovascular risk factors. Given its strong predictive value and biological relevance, suPAR may serve as a promising biomarker for the early detection and risk stratification of ED in diabetes mellitus. Future prospective studies are warranted to validate these findings in larger and more diverse populations and to explore the utility of suPAR-guided interventions in clinical practice.

## Figures and Tables

**Table 1:** The demographic, anthropometric and clinical data of the patients.

	Healthy Control Group 1 (*n* = 46)	DM-NoED Group 2 (*n* = 54)	DMED Group 3 (*n* = 73)	*p*
Age, years	55 (51–61)	57 (49–64)	60 (51–64)	0.055
** Smoking status, *n* (%) **				
- Never	29 (63)	38 (70)	45 (63)	0.583
- Ex-smoker	5 (11)	4 (8)	6 (8)	
- Current smoker	12 (25)	12 (22)	22 (30)	
Body mass index, kg/m^2^	23 (21.7–23.9)	27.7 (25–30.5)	28 (25.8–31)	<0.001 *
Systolic BP, mmHg Diastolic BP, mmHg	120 (118–130)	124 (119–130)	130 (120–130)	0.221
	78 (70–85)	80 (72–85)	80 (70–90)	0.151
Diabetes duration, years	-	7 (3–14)	14 (6–19.5)	<0.001
ED duration, years	-	-	4.1	
IIEF-5 score	23 (22–24)	24 (23–25)	11 (6–15)	<0.001 **
** ED severity, *n* (%) **				
- Mild ED - Mild to moderate ED - Moderate ED - Severe ED	-	-	15 (20.5)	
	-	-	19 (26)	
	-	-	16 (22)	
	-	-	23 (31.5)	
**Comorbid diseases, *n* (%)** - Ischemic heart disease - Hypertension - Hyperlipidemia				
	-	12 (22.6)	18 (24.6)	0.782
	-	23 (43)	40 (54.7)	0.394
	-	19 (35.8)	33 (45.2)	0.674
** Medication, *n* (%) **				
- Metformin	-	43 (79.6)	57 (78)	0.959
- DPP4 inhibitors	-	27 (50)	39 (53)	0.695
- SGLT2 inhibitors	-	24 (44)	39 (53)	0.300
- Sulfonylureas	-	11 (20)	13 (17)	0.681
- Pioglitazone	-	5 (9.2)	12 (16.4)	0.180
- Insulin	-	12 (22)	26 (35.6)	0.072
- ACEI or ARB	-	12 (22.2)	25 (34)	0.103
- Statin and/or fibrate	-	14 (25.9)	30 (41)	0.053

DM-NoED: diabetes mellitus without erectile dysfunction; DMED: diabetes mellitus with erectile dysfunction; BP: blood pressure; ED: erectile dysfunction; IIEF-5: International Index of Erectile Function-5; DPP4: dipeptidyl peptidase 4; SGLT2: sodium–glucose cotransporter 2; ACEI: angiotensin converting enzyme inhibitor; ARB: angiotensin receptor blocker. Data are presented as median (interquartile range). Categorical parameters are expressed as n (percentage). * Post hoc analysis was performed with Dunn’s method. * *p* < 0.001 between Groups 1 and 2 and between groups 1 and 3. ** *p* < 0.001 between Groups 1 and 3 and between groups 2 and 3.

**Table 2:** Comparison of serum suPAR levels and relevant laboratory parameters between groups.

	Healthy Control Group 1 (*n* = 46)	DM-NoED Group 2 (*n* = 54)	DMED Group 3 (*n* = 73)	*p*
suPAR, pg/mL	107.9 (102–116)	130.3 (112–165)	218 (192–249)	<0.001 *
FBG, mg/dL	92 (84–96)	148 (104–245)	175 (130–221)	<0.001 **
HbA1c, %	5.7 (5.4–5.9)	7.8 (6.8–9.6)	8 (7.2–9.3)	<0.001 **
HOMA-IR	2.2 (1.5–3)	4 (2.2–10.3)	4.9 (1.7–8.7)	<0.001 **
LDLc, mg/dL	113 (91–137)	98 (80–133)	109 (80–133)	0.368
HDLc, mg/dL	40 (36–45)	39 (33–43)	41 (34–48)	0.204
TG, mg/dL	150 (107–200)	145 (98–212)	152 (104–234)	0.582
T.testosterone, ng/mL	4.5 (2.9–5.5)	3.4 (2.5–5)	3.8 (2.8–5)	0.131
Total PSA, µg/L	1.2 (0.8–2.7)	1.3 (0.6–2.7)	1.4 (0.5–2.5)	0.413
Prolactin, µg/mL	11 (7.3–14.7)	8.1 (6.8–12)	9.9 (7.2–13.6)	0.187
FSH, IU/L	8.8 (6.7–11.7)	7.7 (5.7–9.4)	8.1 (7–11.4)	0.390
LH, IU/L	6.1 (5–7.9)	6.2 (5.3–7.9)	5.8 (4.7–7.1)	0.508
TSH, mIU/L	1.5 (1–2.9)	1.4 (1.1–2.5)	2.1 (1.4–2.8)	0.106
Leukocytes, 10^3^/uL	7.8 (6.7–8.8)	7.9 (6.4–9.8)	8.3 (6.6–10)	0.330
Hemoglobin, g/dL	14.9 (14.3–15.8)	14.7 (13.6–15.7)	14.8 (14–15.5)	0.085
Platelets, 10^3^/uL	242 (220–264)	257 (212–293)	249 (208–308)	0.881
Creatinine, mg/dL	0.9 (0.8–1)	0.9 (0.8–1)	0.9 (0.6–1.1)	0.664
eGFR, mL/min/1.73 m^2^	93 (84–101)	92 (80–104)	91 (75–100)	0.091
ALT, U/L	20 (14–28)	16 (12–27)	18 (15–23)	0.316
Total protein, g/L	7.5 (7.3–7.7)	7.2 (7.1–7.5)	7.3 (7.2–7.5)	0.490
Albumin, g/L	4.4 (4.2–4.7)	4.5 (4.3–4.7)	4.2 (4–4.7)	0.056
CRP, mg/L	2.1 (1.3–3.4)	2.3 (1.1–4.4)	1.8 (0.9–4.1)	0.623
ESR, mm/h	8 (6–12)	11 (6–17)	9 (6–14)	0.232

DM-NoED: diabetes mellitus without erectile dysfunction; DMED: diabetes mellitus with erectile dysfunction; suPAR: soluble urokinase plasminogen activator receptor; FBG: fasting blood glucose; HbA1c: hemoglobin A1c; HOMA-IR: homeostasis model assessment of insulin resistance; LDLc: low-density-lipoprotein cholesterol; HDLc: high-density-lipoprotein cholesterol; TG: triglyceride; T.testosterone: total testosterone; PSA: prostate-specific antigen; FSH: follicle-stimulating hormone; LH: luteinizing hormone; TSH: thyroid-stimulating hormone; eGFR: estimated glomerular filtration rate; ALT: alanine aminotransferase; CRP: C-reactive protein; ESR: erythrocyte sedimentation rate. * Post hoc analysis was performed with Dunn’s method, and *p* < 0.001 for all groups. ** *p* < 0.001 between Groups 1 and 2 and between groups 1 and 3.

## Data Availability

The data that support the findings of this study are available from the corresponding author upon reasonable request.

## Abbreviations

ED	Erectile dysfunction
DM	Diabetes mellitus
suPAR	Soluble urokinase plasminogen activator receptor
uPAR	Urokinase plasminogen activator receptor
NO	Nitric oxide
PDE5	Phosphodiesterase type 5
IIEF-5	International Index of Erectile Function-5
CRP	C-reactive protein
HbA1c	Glycated hemoglobin
PSA	Prostate-specific antigen
LH	Luteinizing hormone
FSH	Follicle-stimulating hormone
TSH	Thyroid-stimulating hormone
ROC	Receiver operating characteristic
IQR	Interquartile range
DM-NoED	Diabetic patients without erectile dysfunction
DMED	Diabetic patients with erectile dysfunction
ESR	Erythrocyte sedimentation rate
AUC	Area under the curve
